# National and subnational hypertension prevalence estimates for the Republic of Ireland: better outcome and risk factor data are needed to produce better prevalence estimates

**DOI:** 10.1186/1471-2458-14-24

**Published:** 2014-01-10

**Authors:** Steve Barron, Kevin Balanda, John Hughes, Lorraine Fahy

**Affiliations:** 1 Institute of Public Health in Ireland, 5th Floor, Bishop’s Square, Redmond’s Hill, Dublin 2, Ireland; 2 UKCRC Centre of Excellence for Public Health, Centre for Public Health, School of Medicine, Dentistry and Biomedical Sciences, Queen’s University, Belfast, UK

**Keywords:** Hypertension prevalence diagnosed undiagnosed Ireland

## Abstract

**Background:**

Hypertension is a global public health challenge. National prevalence estimates can conceal important differences in prevalence in subnational areas. This paper aims to develop a consistent set of national and subnational estimates of the prevalence of hypertension in a country with limited data for subnational areas.

**Methods:**

A nationally representative cross-sectional Survey of Lifestyle, Attitudes and Nutrition (SLÁN) 2007 was used to identify risk factors and develop a national and a subnational model of the risk of self-reported, doctor-diagnosed hypertension among adults aged 18+ years in the Republic of Ireland. The subnational model’s group-specific risk estimates were applied to group-specific population count estimates for subnational areas to estimate the number of adults with doctor-diagnosed hypertension in subnational areas in 2007. A sub-sample of older adults aged 45+ years who also had their blood pressure objectively measured using a sphygmomanometer was used to estimate the national prevalence of diagnosed and undiagnosed hypertension among adults aged 45+ years.

**Results:**

The prevalence of self-reported, doctor-diagnosed hypertension among adults aged 18+ years was 12.6% (95% CI = 11.7% - 13.4%). After adjustment for other explanatory variables the risk of self-reported, doctor-diagnosed hypertension was significantly related to age (p < 0.001), body mass index (p < 0.001), smoking (p = 0.001) and fruit and vegetable consumption (p = 0.003). Among adults aged 45+ years the prevalence of undiagnosed hypertension (38.7% (95% CI 34.6% - 42.8%)) was higher than self-reported, doctor-diagnosed hypertension (23.4% (95% CI = 22.0% - 24.7%)). Among adults aged 45+ years, the prevalence of undiagnosed hypertension was higher among men (46.8%, 95% CI 41.2% - 52.4%) than women (31.2%, 95% CI 25.7% - 36.6%). There was no significant variation in the prevalence of self-reported, doctor-diagnosed hypertension across subnational areas.

**Conclusions:**

Services need to manage diagnosed hypertension cases and to detect and manage undiagnosed cases. Further population level improvements in lifestyle risk factors for hypertension are key in developing a more integrated approach to prevent cardiovascular disease. Better subnational data on hypertension outcomes and risk factors are needed to better describe the distribution of hypertension risk and hypertension prevalence in subnational areas.

## Background

Hypertension is a global public health challenge [1,2] responsible for significantly reduced quality of life and is a risk factor strongly associated with cardiovascular disease and premature mortality [3-5]. The prevalence of hypertension is expected to increase as the population increases and ages [6].

Population prevalence of hypertension is the proportion of people living with hypertension in a population at or during a particular time period. National prevalence can conceal important differences in prevalence in subnational areas [7]. Subnational estimates of the population prevalence of hypertension are essential for the development of healthy and equitable communities. Subnational estimates describe the pattern of disease in the local population and support the planning and delivery of prevention and management services that meet local needs.

Estimates of the prevalence of hypertension in the Republic of Ireland range from 10% [8] to 62% [9] depending on what outcomes, age groups and methods were used. There is no consistent approach based on the national health survey that provides national and subnational prevalence estimates.

The Institute of Public Health in Ireland (IPH) has produced national and subnational prevalence estimates and forecasts for a number of chronic health conditions. We first published diabetes prevalence estimates and forecasts for the Republic of Ireland and Northern Ireland [10,11] based on a prevalence model developed in England by the Association of Public Health Observatories (APHO) [12]. This model used diabetes risks from an English population-based epidemiological study applied to population data from the island of Ireland. We then adapted other APHO disease prevalence models [12] based on the Health Survey for England to estimate and forecast the prevalence of hypertension, stroke, coronary heart disease and chronic airflow obstruction on the island of Ireland [13,14]. Due to concerns about using English data to estimate disease risks for the island of Ireland, we further adapted the methodology for use with Republic of Ireland and Northern Ireland data. Prevalence estimates and forecasts based on this method and Irish data sources have been published elsewhere [15,16].

This paper describes the method in detail and uses it to produce a consistent set of national and subnational estimates of the prevalence of hypertension for the Republic of Ireland. There are a number of significant limitations in the subnational risk factor data available in the Republic of Ireland. These limitations resulted in a number of compromises in our efforts to develop a systematic and rigorous methodology. This paper describes these data limitations and how risk factor data could be improved to produce better prevalence estimates.

## Methods

### National model of hypertension risk

#### Data sources

The Survey of Lifestyle, Attitudes and Nutrition (SLÁN) 2007 [9], a cross-sectional survey of health and lifestyle, was used to describe the risk of hypertension in the Republic of Ireland. A nationally representative sample of private households was selected from the country’s GeoDirectory [17], the Irish address database. One adult within a household was randomly selected to participate in the survey. The survey comprised face-to-face detailed health and lifestyle interviews with 10,364 respondents aged 18+ years administered by trained social interviewers as well as two physical measurement sub-samples: anthropometric measures in a sub-sample of 967 respondents aged 18-44 years and a more detailed physical examination of a sub-sample of 1,207 respondents aged 45+ years. The response rate for the main survey of adults aged 18+ years was 62% (n = 10,364). The response rate within these respondents to the main survey was 58% (n = 967) for the younger sub-sample (aged 18-44 years) and 60% (n = 1,207) for the older sub-sample (aged 45+ years). The full sample distribution and the sub-sample distributions were weighted to population totals (for age, gender, marital status, economic status, education, occupational category, ethnicity, household size, and geographic region) using the Quarterly National Household Survey (QNHS) [18] and Census 2006 [19].

Census 2006 population counts were obtained from the Central Statistics Office and were adjusted for differences to population between Census 2006 and population count estimates 2007.

### Measurements

All survey respondents were asked if, in the last 12 months, they had been told by a doctor that they had high blood pressure (yes/no). Respondents in the older sub-sample (aged 45+ years) were also asked if they were currently taking medication for high blood pressure (yes/no) and had their blood pressure objectively measured. Resting, sitting blood pressure was measured in the right upper arm using an OMRON 705IT Digital Automatic Blood Pressure Monitor. Three readings were measured per respondent and the mean of the second and third readings were used for analysis. This older sub-sample (aged 45+ years) was used to estimate the prevalence of undiagnosed hypertension amongst those aged 45+ years. Respondents were classified as having undiagnosed hypertension based on the following criteria: i) they did not self-report a doctor diagnosis in the past 12 months, and ii) they had physically measured hypertension (≥140 mmHg SBP or ≥90 mmHg DBP) and/or were using anti-hypertensive medication. Population prevalence of hypertension (diagnosed and undiagnosed) amongst adults aged 45+ years was taken to be the sum of the doctor-diagnosed rate and the undiagnosed rate.

Table 1 shows the socio-demographic and lifestyle variables that were available for all respondents. These were the potential explanatory variables for a statistical model of self-reported, doctor-diagnosed hypertension. Actual height, weight and waist circumference were measured for the 2,170 respondents in the younger sub-sample (aged 18-44 years) and the older sub-sample (aged 45+ years). BMI was calculated for the remaining respondents by multiplying self-reported weight and height by an adjustment factor which ensured that, in each sex-age category, the mean of the adjusted self-reported BMIs matched the mean measured BMI in the associated sub-sample. The adjustment factors were larger for females and older respondents, ranging from 1.03 to 1.08.

**Table 1:** Explanatory variables included in the stepwise selection algorithm for self-reported, doctor-diagnosed hypertension

**Explanatory variable**	**Categories**
Sex	Male
	Female
Age	18-34 years
	35-44 years
	45-54 years
	55-64 years
	65-74 years
	75+ years
Ethnicity	White
	Non-white
Body mass index (BMI)	Underweight/Normal (BMI < 25)
	Overweight (BMI 25-29.9)
	Obese (BMI 30+)
	Low
Physical activity (based on the International Physical Activity Questionnaire (IPAQ))	Moderate
	High
Cigarette smoking	Never smoked
	Former smoker
	Current smoker
Alcohol consumption	Never/monthly or less/2-4 times per month
	2-3 times per week
	4+ times per week
Fruit and vegetable consumption	< 5 portions per day
	5+ portions per day
Salt use while cooking	Always/usually, sometimes
	Sometimes
	Rarely/never
Salt use at the table	Always/usually, sometimes
	Sometimes
	Rarely/never
Highest level of education	Primary level
	Secondary level
	Third level
Employment status	Employed
	Unemployed
	Economically inactive
Social class	SC 1-2 (Professional and managerial)
	SC 3-4 (Non-manual and skilled manual)
	SC 5-6 (Semi-skilled and unskilled)
	Unclassified
Area deprivation [20]	Quintiles

### Statistical modelling

All models were developed using weighted data. Self-reported, doctor-diagnosed hypertension (rather than physically measured hypertension) was chosen as the model outcome because it was available for all adult age groups (18+ years compared with 45+ years) and with a larger sample (10,201 respondents aged 18+ years compared with 1,201 respondents aged 45+ years).

**Figure 1:**
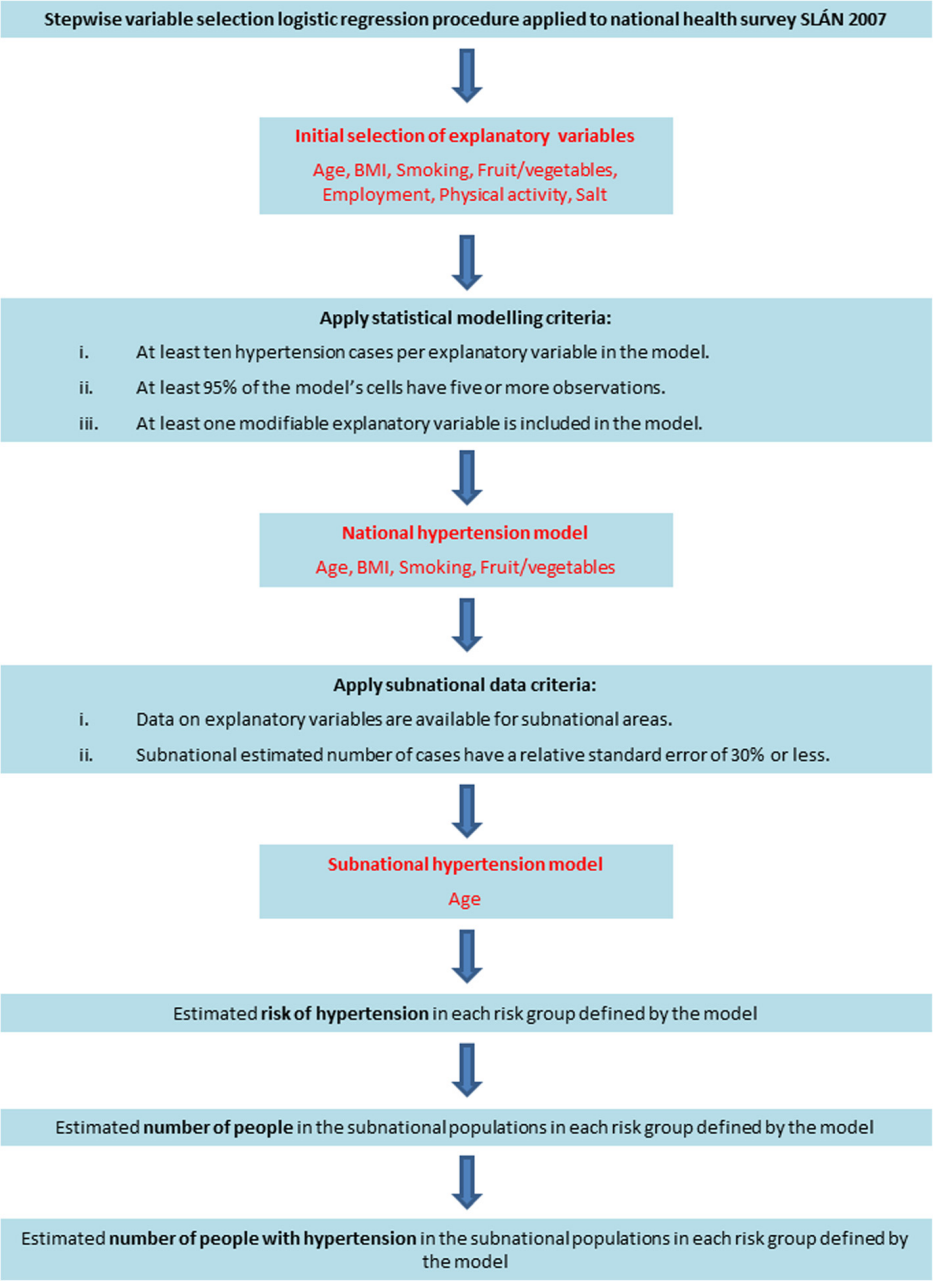
Method for estimating the national and subnational prevalence of self-reported, doctor-diagnosed hypertension.

A stepwise selection logistic regression procedure [21] was used to develop a statistical model of this outcome (see Figure 1). The selection procedure (SAS Version 9.2 PROC LOGISTIC, entry p-value = 0.05, exit p-value = 0.05) [22] identified which of the socio-demographic and lifestyle variables listed in Tale 1 were multvariably associated with doctor-diagnosed hypertension. The initial selection of explanatory variables consisted of age, BMI, smoking, fruit and vegetable consumption, employment status, physical activity and salt use at the table.

A national model was developed from the initial selection of explanatory variables. The national model was required to satisfy a number of statistical modelling criteria: i) at least ten hypertension cases per explanatory variable in the model [23], ii) at least 95% of the model’s cells had five or more observations [24], iii) at least one modifiable explanatory variable in the model. To satisfy criterion ii) it was necessary to reduce the number of cells in the initial selection of explanatory variables so that a sufficient number of cells had a sufficient number of observations. The number of cells in the initial selection of explanatory variables was reduced by removing weaker explanatory variables (based on the Chi-Square statistic adjusted for other explanatory variables included in the model) until criterion ii) was satisfied. The weaker explanatory variables removed from the initial selection of explanatory variables were employment status, physical activity and salt use at the table. The remaining explanatory variables (age, BMI, smoking, fruit and vegetable consumption) were then refitted as a national model that satisfied all three statistical modelling criteria. The model was refit using PROC SURVEYLOGISTIC which takes into account geographical clustering in the sample design. The model divided the population into risk groups defined by the categories of the explanatory variables and provided an estimate of the risk (at national level) of having doctor-diagnosed hypertension in each of these risk groups.

### Subnational prevalence

#### Developing a subnational model of hypertension risk

Health services in the Republic of Ireland are delivered by the Health Service Executive through 32 Local Health Offices (LHOs). Two criteria were used to modify the national model to obtain a subnational model that could be used to estimate prevalence for these subnational LHOs. These criteria were: i) data on the explanatory variables needed to be available for LHOs so that the number of people in each of the risk groups in LHOs could be estimated, and ii) the prevalence estimates for LHOs needed to be satisfactorily precise such that all LHOs’ estimated number of cases had a relative standard error of 30% or less (that is, the standard error of the estimate was no more than 30 per cent of the estimate) [25].

Age was the only explanatory variable in the national model for which data were available for LHOs.

Data on the other explanatory variables (smoking, BMI, fruit and vegetable consumption) were not available for LHOs and these explanatory variables were removed from the model. A subnational model consisting of only age was refit using the same observations as the national model so that the national and subnational models would produce consistent prevalence estimates.

### Constructing the subnational risk group populations

LHO population counts by age and sex were obtained from Census 2006. Population count estimates are not routinely produced for LHOs but are produced for eight larger subnational areas (Regional Authorities) [26]. Age-sex specific changes in population between Census 2006 and 2007 population estimates were calculated for each Regional Authority. These Regional Authority adjustment factors were applied to age-sex specific LHO population counts from Census 2006 to approximate 2007 LHO population estimates by age.

### Calculating the estimated number of cases in the subnational populations

The subnational model’s age group risk estimates were multiplied by the corresponding LHO age group population count estimates to estimate the number of adults with doctor-diagnosed hypertension in 2007. The age-specific estimated number of cases and prevalence rates from the model were scaled to be consistent with the age-specific prevalence rates from the SLÁN survey without modelling.

In calculating 95% confidence intervals for the prevalence estimates we assumed that: i) the age group risk estimates and the age group population count estimates in each LHO were statistically independent, and ii) the number of people in each age group in each LHO in the population was known without error.

### Ethics statement

This study did not require any ethical approval as it involved secondary analyses of publicly available demographic and population data.

## Results

### Risk of doctor-diagnosed hypertension amongst adults aged 18+ years

The national model derived from the stepwise regression procedure explained significant deviance (Likelihood Ratio Chi-squared test = 1086.70, df = 10, p < 0.001) and showed no evidence of lack of fit (Hosmer and Lemeshow Chi-Square test = 9.02, df = 8, p = 0.34). The area under the ROC curve of 0.7729 (95% CI = 0.7594 - 0.7863) suggests that the national model’s performance at separating cases and non-case was “fair.” The explanatory variables in the model were age, BMI, smoking, and fruit and vegetable consumption. After adjustment for all other explanatory variables the risk of self-reported, doctor-diagnosed hypertension was significantly related to age (Chi-Square = 424.10, df = 5, p < 0.001), BMI (Chi-Square = 114.74, df = 2, p < 0.001), smoking (Chi-Square = 13.11, df = 2, p = 0.001) and fruit and vegetable consumption (Chi-Square = 8.62, df = 1, p = 0.003).

Table 2 identifies subgroups of respondents who were at significantly increased independent risk of self-reported, doctor-diagnosed hypertension. After adjustment for the other explanatory variables all age groups were more likely than those aged 18-34 years to have doctor-diagnosed hypertension, overweight and obese respondents were more likely than underweight/normal weight respondents to have doctor-diagnosed hypertension, current smokers and former smokers were more likely than people who never smoked to have doctor-diagnosed hypertension, and respondents who ate less fruit and vegetables were more likely to have doctor-diagnosed hypertension.

**Table 2:** Odds ratios for the national model of self-reported, doctor-diagnosed hypertension

		**Odds ratio (OR)**	**95% CI for OR**	**Pr > ChiSq**
Age	18-34 years (reference)	1.00		
	35-44 years	2.02	1.42 - 2.87	<.0001
	45-54 years	5.46	3.95 - 7.54	<.0001
	55-64 years	10.68	7.75 - 14.73	<.0001
	65-74 years	12.92	9.04 - 18.46	<.0001
	75+ years	14.45	9.77 - 21.40	<.0001
BMI	Underweight/normal (reference)	1.00		
	Overweight 25-29.99	1.57	1.27 - 1.95	<.0001
	Obese 30+	3.20	2.57 - 3.98	<.0001
Smoking	Never smoked (reference)	1.00		
	Current smoker	1.28	1.04 - 1.58	0.0209
	Former smoker	1.40	1.15 - 1.70	0.0007
Fruit and vegetables	5 or more portions (reference)	1.00		
	Less than 5 portions	1.30	1.09 - 1.56	0.0033

### Prevalence of doctor-diagnosed hypertension amongst adults aged 18+ years

**Figure 2:**
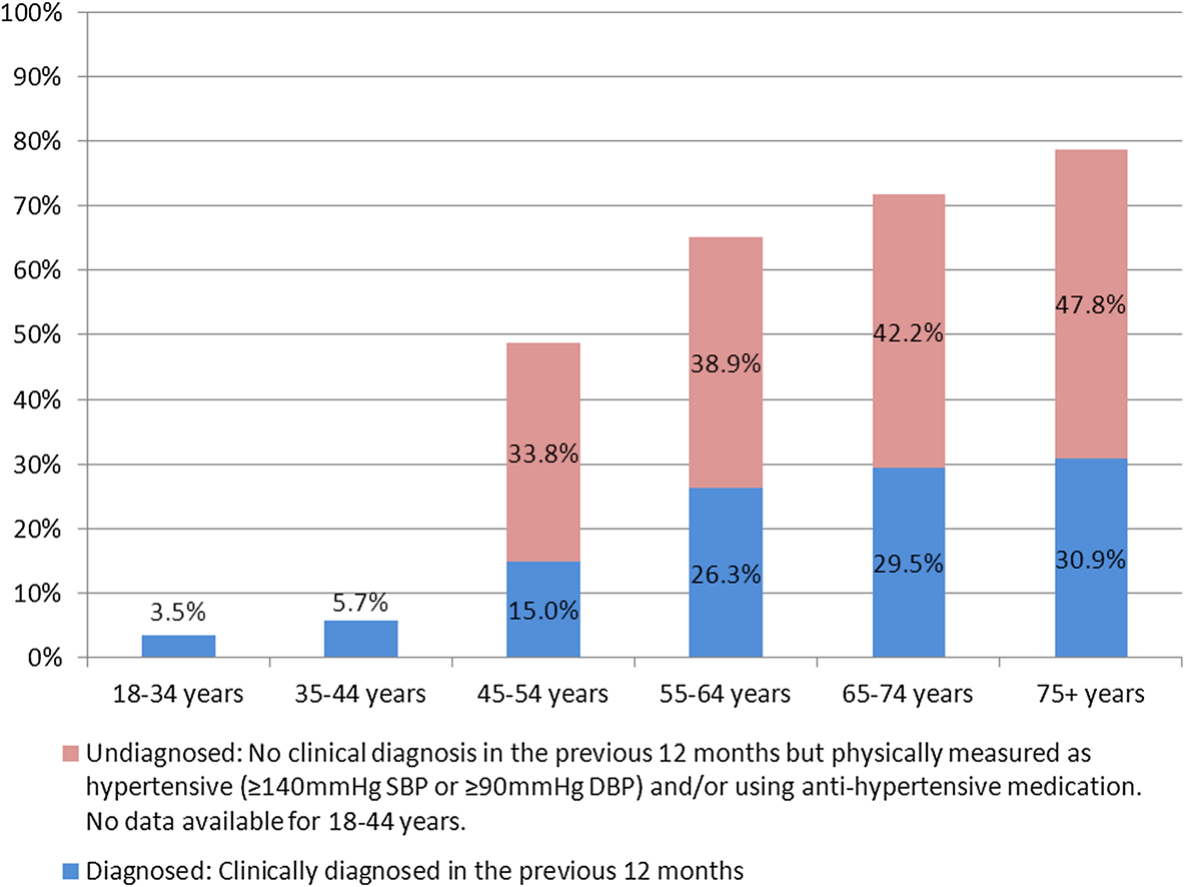
Prevalence of self-reported, doctor-diagnosed hypertension and undiagnosed hypertension (Republic of Ireland, 2007).

It was estimated that 12.6% (95% CI = 11.7% - 13.4%) of adults aged 18+ years in the Republic of Ireland had doctor-diagnosed hypertension in 2007. The prevalence of doctor-diagnosed hypertension was similar amongst males and females aged 18-44 years (p = 0.96) and males and females aged 45+ years (p = 0.25). Doctor-diagnosed hypertension was significantly more common among older respondents (see Figure 2).

### Prevalence of doctor-diagnosed and undiagnosed hypertension amongst adults aged 45+ years

Table 3 describes the prevalence of doctor-diagnosed and undiagnosed hypertension amongst adults aged 45+ years. The prevalence of undiagnosed hypertension was significantly higher than diagnosed hypertension for both men and women aged 45+ years. The prevalence of undiagnosed hypertension was significantly higher among men aged 45+ years (46.8%, 95% CI 41.2% - 52.4%) than women aged 45+ years (31.2%, 95% CI 25.7% - 36.6%).

**Table 3:** **Prevalence of hypertension by sex and age**^**1**^

	**Total (diagnosed & undiagnosed)**	**Doctor-diagnosed**	**Undiagnosed**	**Percentage of cases undiagnosed**
	**(95% CI)**	**(95% CI)**^2^	**(95% CI)**^3^	
**Sex**				
Males	71.0% (65.1% - 77.0%)	24.2% (22.2% - 26.3%)	46.8% (41.2% - 52.4%)	65.9%
Females	53.7% (48.0% - 59.4%)	22.5% (20.6% - 24.5%)	31.2% (25.7% - 36.6%)	58.0%
**Age (persons)**				
45+ years	62.0% (57.8% - 66.3%)	23.4% (22.0% - 24.7%)	38.7% (34.6% - 42.8%)	62.4%
45-54 years	48.8% (42.5% - 55.0%)	15.0% (12.9% - 17.0%)	33.8% (27.8% - 39.8%)	69.3%
55-64 years	65.1% (57.0% - 73.2%)	26.3% (23.5% - 29.1%)	38.9% (31.2% - 46.5%)	59.7%
65–74 years	71.8% (62.6% - 80.9%)	29.5% (26.2% - 32.9%)	42.2% (33.7% - 50.8%)	58.8%
75+ years	78.7% (63.0% - 94.5%)	30.9% (26.2% - 35.6%)	47.8% (32.7% - 62.9%)	60.8%
**Age (males)**				
45+ years	71.0% (65.1% - 77.0%)	24.2% (22.2% - 26.3%)	46.8% (41.2% - 52.4%)	65.9%
45-54 years	61.1% (51.5% - 70.6%)	16.2% (13.0% - 19.5%)	44.8% (35.8% - 53.8%)	73.4%
55-64 years	70.1% (58.2% - 82.0%)	28.3% (24.0% - 32.5%)	41.8% (30.7% - 53.0%)	59.7%
65–74 years	83.9% (72.4% - 95.5%)	31.5% (27.1% - 36.0%)	52.4% (41.7% - 63.1%)	62.4%
75+ years	86.5% (68.0% - 105.1%)	27.8% (22.1% - 33.4%)	58.8% (41.0% - 76.6%)	67.9%
**Age (females)**				
45+ years	53.7% (48.0% - 59.4%)	22.5% (20.6% - 24.5%)	31.2% (25.7% - 36.6%)	58.0%
45-54 years	36.5% (28.6% - 44.3%)	13.7% (11.1% - 16.3%)	22.7% (15.3% - 30.2%)	62.4%
55-64 years	60.3% (49.9% - 70.6%)	24.3% (20.6% - 28.1%)	35.9% (26.3% - 45.6%)	59.6%
65–74 years	60.8% (47.6% - 74.0%)	27.7% (22.6% - 32.9%)	33.1% (20.9% - 45.3%)	54.4%
75+ years	73.6% (52.3% - 95.0%)	32.9% (26.5% - 39.4%)	40.7% (20.3% - 61.1%)	55.3%

^1^Some column totals will differ from sum of corresponding entries because of rounding error.

^2^Based on the full sample.

^3^Based on the older physical measurement sub-sample only.

### Subnational variation in prevalence of doctor-diagnosed hypertension amongst adults aged 18+ years

The area under the ROC curve of the subnational model (0.7508, 95% CI = 0.7374 - 0.7643) was not significantly different to that of the national model (0.7729, 95% CI = 0.7594 - 0.7863). These classification statistics are similar because both the national and subnational models include the strongest predictor of diagnosed hypertension – age.

**Figure 3:**
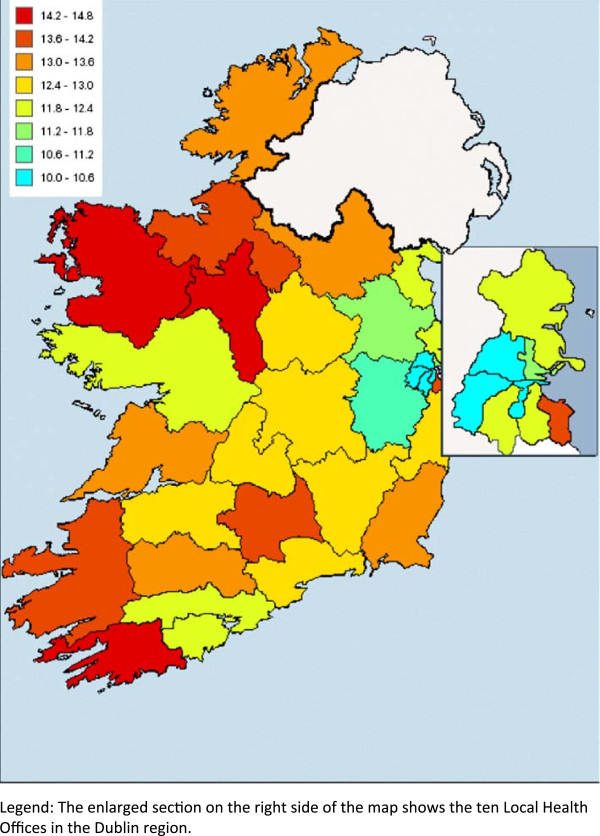
Prevalence of self-reported, doctor-diagnosed hypertension among adults by Local Health Offices (Republic of Ireland, 2007).

Figure 3 shows the subnational variation in the prevalence of doctor-diagnosed hypertension amongst adults aged 18+ years. Although the national model included four explanatory variables (age, BMI, smoking, and fruit and vegetable consumption), the subnational model only included age because age was the only explanatory variable for which data were available at LHO level. Therefore, LHO variation in prevalence was determined only by differences in the age structure of the LHO populations.

Pairwise comparisons of 95% confidence intervals revealed no statistically significant variation in the prevalence of doctor-diagnosed hypertension across the LHOs. However, because the LHOs have different adult population sizes, there was substantial variation in the number of adults aged 18+ years in each LHO who had doctor-diagnosed hypertension.

## Discussion

### Summary of findings

This paper describes a systematic and rigorous approach to develop a consistent set of national and subnational estimates of the prevalence of hypertension in the Republic of Ireland, a country with inadequate national and subnational outcome and risk factor data. The method described here (see Figure 1) builds on methods developed by the APHO [10] and has a number of advantages. It is systematic and can be implemented for any condition for which we have a good quality reference study to model the risk of the condition. It is also flexible and could incorporate better input data (reference study and/or population data) if and when they are available.

The prevalence of self-reported, doctor-diagnosed hypertension in the previous 12 months found here (12.6% among adults aged 18+ years) was similar to other surveys from the Republic of Ireland. The QNHS’s prevalence of self-reported, doctor-diagnosed hypertension at any time in the past among adults aged 18+ years was 10% in 2007 [8] and 11% in 2010 [27]. In Northern Ireland among adults aged 16+ years, Understanding Society 2009 [28] reported similar prevalence (15% at any time in the past) but the Health and Social Wellbeing Survey 2005/2006 [29] reported a higher prevalence (25% at any time in the past). The explanatory model of doctor-diagnosed hypertension showed that older adults, overweight and obese adults, current and former smokers, and adults who eat less fruit and vegetables were more likely to have doctor-diagnosed hypertension. There was no statistically significant variation in the prevalence of doctor-diagnosed hypertension amongst adults across the subnational areas.

In our study, the total prevalence of hypertension (diagnosed and undiagnosed; 62% among adults aged 45+ years) included people who had physically measured hypertension or who reported a doctor diagnosis of hypertension in the previous 12 months. This more inclusive definition contributed to a higher estimated prevalence than estimates from other national and international surveys based on physical measurement alone. The Irish Longitudinal Study on Ageing (TILDA) estimated that 43% of adults aged 50+ years have physically measured hypertension [30] but this estimate excluded people with hypertension controlled by medication. The Health Survey for England 2011 estimated that 47% of adults aged 45+ years hade physically measured hypertension [31]. In the US the National Health and Nutrition Examination Survey (NHANES) 2009/2010 estimated that 30% of 40-59 years olds and 67% of 60+ year olds had physically measured hypertension [32].

Amongst the group aged 45+ years the majority of cases (62%) were undiagnosed and men were more likely than women to have undiagnosed hypertension. TILDA also found that a slight majority of cases were undiagnosed in the Republic of Ireland (53% of cases among 50+ year olds). The HSfE 2011 found that a third of cases among 35+ year olds in England were undiagnosed while NHANES 2009/2010 found that only one sixth of cases among 40+ year olds in the US were undiagnosed.

### Limitations

There were a number of limitations to SLÁN 2007 for modelling the risk of hypertension and to the population data on hypertension risk factors.

The hypertension outcome used in the explanatory model of SLÁN 2007 data was self-reported, doctor-diagnosed hypertension in the previous 12 months because it was available for the full sample of adults aged 18+ years. Physically measured blood pressure was only available for a smaller sub-sample of older adults aged 45+ years. The findings from the older sub-sample, where the significant majority of hypertension was undiagnosed, showed that self-reported, doctor-diagnosed hypertension was a substantial underestimate of the population prevalence of hypertension.

Inadequate sample size contributed to an insufficient number of observations in the risk groups of the initial selection of explanatory variables and this may bias the risk estimates produced from such a model. A model with fewer explanatory variables (and therefore fewer risk groups and more observations in each risk group) may reduce bias in the risk estimates. Three weaker significant explanatory variables were removed from the initial selection to achieve adequate sample sizes in the risk groups for the national model. Although this may reduce bias in the risk estimates, it also reduced the model’s goodness of fit.

Inadequate sample size and a sampling design that was not representative at subnational areas prohibited the development of different models for different subnational areas.

The response rate for the main survey was 62%. The response rate within these respondents to the main survey was 58% for the younger sub-sample and 60% for the older sub-sample.

In addition to the limitations to the survey data for modelling the risk of hypertension, there were substantial limitations to the data available to describe the distribution of risk in subnational populations. Population count estimates by age are routinely produced for eight Regional Authority areas but not for smaller LHO areas. Data on behavioural and social risk factors for hypertension were not available for LHOs and these factors were removed when specifying the subnational model. These limitations prevented us from properly describing the distribution of risk in LHOs and were likely to mask LHO variation in the prevalence of hypertension.

## Conclusions and recommendations

The findings suggest that a large number of adults in the Republic of Ireland are living with hypertension and that the majority of cases are undiagnosed. They highlight the need for services to manage diagnosed hypertension cases and to detect and manage undiagnosed cases. Symptoms of hypertension are often hidden and people often do not know that they have hypertension until their blood pressure is measured. Regular blood pressure measurement in primary care allows early detection and treatment of hypertension that can avoid further damage to health. The Republic of Ireland’s national cardiovascular health policy [33] recommends that the effective management of hypertension should be prioritised in primary care and calls for guidelines on standards of assessment, management and review of patients based on best practice.

It is important to emphasise that hypertension can be prevented and that population measures such as mandatory and voluntary reductions of salt content in processed foods are more cost effective than treatment [34]. Individuals should be educated about the dangers of hypertension and encouraged to reduce their risk of hypertension by having a healthy lifestyle: eating a balanced diet, maintaining a healthy weight, reducing salt intake, not smoking, and avoiding harmful use of alcohol. The World Health Organization (WHO) [35], the Republic of Ireland’s national cardiovascular health policy [33], and the Republic of Ireland’s framework for improved health and wellbeing [36] identify population level improvements in these risk factors for hypertension as key in an integrated approach to preventing cardiovascular disease.

The WHO also highlights the importance of collecting reliable data on cardiovascular risk factors and their determinants for policy and programme development [2]. This study found that there are substantial limitations to the data available on hypertension and its risk factors. The following recommendations would improve the data available for monitoring the prevalence of hypertension and its risk factors: i) national health surveys should include physical measurements of blood pressure in all participants in addition to self-reported recall of a hypertension diagnosis, ii) larger sample sizes in these studies would allow more robust and precise risk estimates and could also allow the development of different prevalence models for different subnational areas, iii) higher response rates may reduce potential sampling bias and produce more accurate results, and iv) better subnational data on behavioural and social risk factors for hypertension would allow a better description of the distribution of hypertension risk and hypertension prevalence in subnational areas.

## Competing interests

The authors declare that they have no competing interests.

## Authors’ contributions

KB & SB conceived the original idea for the study. All authors contributed to the development of the study methodology. SB and JH conducted the analysis. All authors contributed to the writing and approved the manuscript. SB is the corresponding author.

## Pre-publication history

The pre-publication history for this paper can be accessed here:

http://www.biomedcentral.com/1471-2458/14/24/prepub
